# Job Satisfaction, Organizational Commitment and Job Involvement: The Mediating Role of Job Involvement

**DOI:** 10.3389/fpsyg.2018.00132

**Published:** 2018-02-16

**Authors:** Jelena Ćulibrk, Milan Delić, Slavica Mitrović, Dubravko Ćulibrk

**Affiliations:** Department of Industrial Engineering and Management, Faculty of Technical Sciences, University of Novi Sad, Novi Sad, Serbia

**Keywords:** work, job, satisfaction, involvement, employee, commitment, organizational, behavior

## Abstract

We conducted an empirical study aimed at identifying and quantifying the relationship between work characteristics, organizational commitment, job satisfaction, job involvement and organizational policies and procedures in the transition economy of Serbia, South Eastern Europe. The study, which included 566 persons, employed by 8 companies, revealed that existing models of work motivation need to be adapted to fit the empirical data, resulting in a revised research model elaborated in the paper. In the proposed model, job involvement partially mediates the effect of job satisfaction on organizational commitment. Job satisfaction in Serbia is affected by work characteristics but, contrary to many studies conducted in developed economies, organizational policies and procedures do not seem significantly affect employee satisfaction.

## 1. Introduction

In the current climate of turbulent changes, companies have begun to realize that the employees represent their most valuable asset (Glen, 2006; Govaerts et al., 2011; Fulmer and Ployhart, 2014; Vomberg et al., 2015; Millar et al., 2017). Satisfied and motivated employees are imperative for contemporary business and a key factor that separates successful companies from the alternative. When considering job satisfaction and work motivation in general, of particular interest are the distinctive traits of these concepts in transition economies.

Serbia is a country that finds itself at the center of the South East region of Europe (SEE), which is still in the state of transition. Here transition refers to the generally accepted concept, which implies economic and political changes introduced by former socialist countries in Europe and beyond (e.g., China) after the years of economic stagnation and recession in the 1980's, in the attempt to move their economy from centralized to market-oriented principles (Ratkovic-Njegovan and Grubic-Nesic, 2015). Serbia exemplifies many of the problems faced by the SEE region as a whole, but also faces a number of problems uniquely related to the legacy of its past. Due to international economic sanctions, the country was isolated for most of the 1990s, and NATO air strikes, related to the Kosovo conflict and carried out in 1999, caused significant damage to the industry and economy. Transitioning to democracy in October 2000, Serbia embarked on a period of economic recovery, helped by the introduction of long overdue reforms, major inflows of foreign investment and substantial assistance from international funding institutions and others in the international community. However, the growth model on which Serbia and other SEE countries relied between 2001 and 2008, being based mainly on rapid capital inflows, a credit-fueled domestic demand boom and high current account deficit (above 20% of GDP in 2008), was not accompanied by the necessary progress in structural and institutional reforms to make this model sustainable (Uvalic, 2013). The central issue of the transition process in Serbia and other such countries is privatization of public enterprises, which in Serbia ran slowly and with a number of interruptions, failures and restarts (Radun et al., 2015). The process led the Serbian industry into a state of industrial collapse, i.e., deindustrialization. Today there are less than 400,000 employees working in the industry in Serbia and the overall unemployment rate exceeds 26% (Milisavljevic et al., 2013). The average growth of Serbia's GDP in the last 5 years was very low, at 0.6% per year, but has reached 2.7% in 2016 (GDP, 2017). The structure of the GDP by sector in 2015 was: services 60.5%, industry 31.4%, and agriculture 8.2% (Statistical Office of the Republic of Serbia, 2017).

Taking into account the specific adversities faced by businesses in Serbia, we formulated two main research questions as a starting point for the analysis of the problem of work motivation in Serbia:

1. To what extent are the previously developed models of work motivation (such as the model of Locke and Latham, 2004) applicable to the transition economy and business practices in Serbia?
2. What is the nature of the relationships between different segments of work motivation (job satisfaction, organizational commitment, job involvement and work characteristics)?

The Hawthorn experiment, conducted in early 1930s (Mayo, 1933), spurred the interest of organizational behavior researchers into the problem of work motivation. Although Hawthorn focused mainly on the problems of increasing the productivity and the effects of supervision, incentives and the changing work conditions, his study had significant repercussions on the research of work motivation. All modern theories of work motivation stem from his study.

Building on his work, Maslow (1943) published his Hierarchy of Needs theory, which remains to this day the most cited and well known of all work motivation theories according to Denhardt et al. (2012). Maslow's theory is a *content-based theory*, belonging to a group of approaches which also includes the ERG Theory by Alderfer (1969), the Achievement Motivation Theory, Motivation-Hygiene Theory and the Role Motivation Theory.

These theories focus on attempting to uncover what the needs and motives that cause people to act in a certain way, within the organization, are. They do not concern themselves with the process humans use to fulfill their needs, but attempt to identify variables which influence this fulfillment. Thus, these theories are often referred to as *individual theories*, as they ignore the organizational aspects of work motivation, such as job characteristics or working environment, but concentrate on the individual and the influence of an individual's needs on work motivation.

The approach is contrasted by the *process theories* of work motivation, which take the view that the concept of needs is not enough to explain the studied phenomenon and include expectations, values, perception, as important aspects needed to explain why people behave in certain ways and why they are willing to invest effort to achieve their goals. The process theories include: Theory of Work and Motivation (Vroom, 1964), Goal Setting Theory (Locke, 1968), Equity Theory (Adams, 1963), as well as the The Porter-Lawler Model (Porter and Lawler, 1968).

Each of these theories has its limitations and, while they do not contradict each other, they focus on different aspects of the motivation process. This is the reason why lately they have been several attempts to create an integrated theory of work motivation, which would encompass all the relevant elements of different basic theories and explain most processes taking place within the domain of work motivation, the process of motivation, as well as employee expectations (Donovan, 2001; Mitchell and Daniels, 2002; Locke and Latham, 2004). One of the most influential integrated theories is the theory proposed by Locke and Latham (2004), which represents the basis for the study presented in this paper.

**Figure 1:**
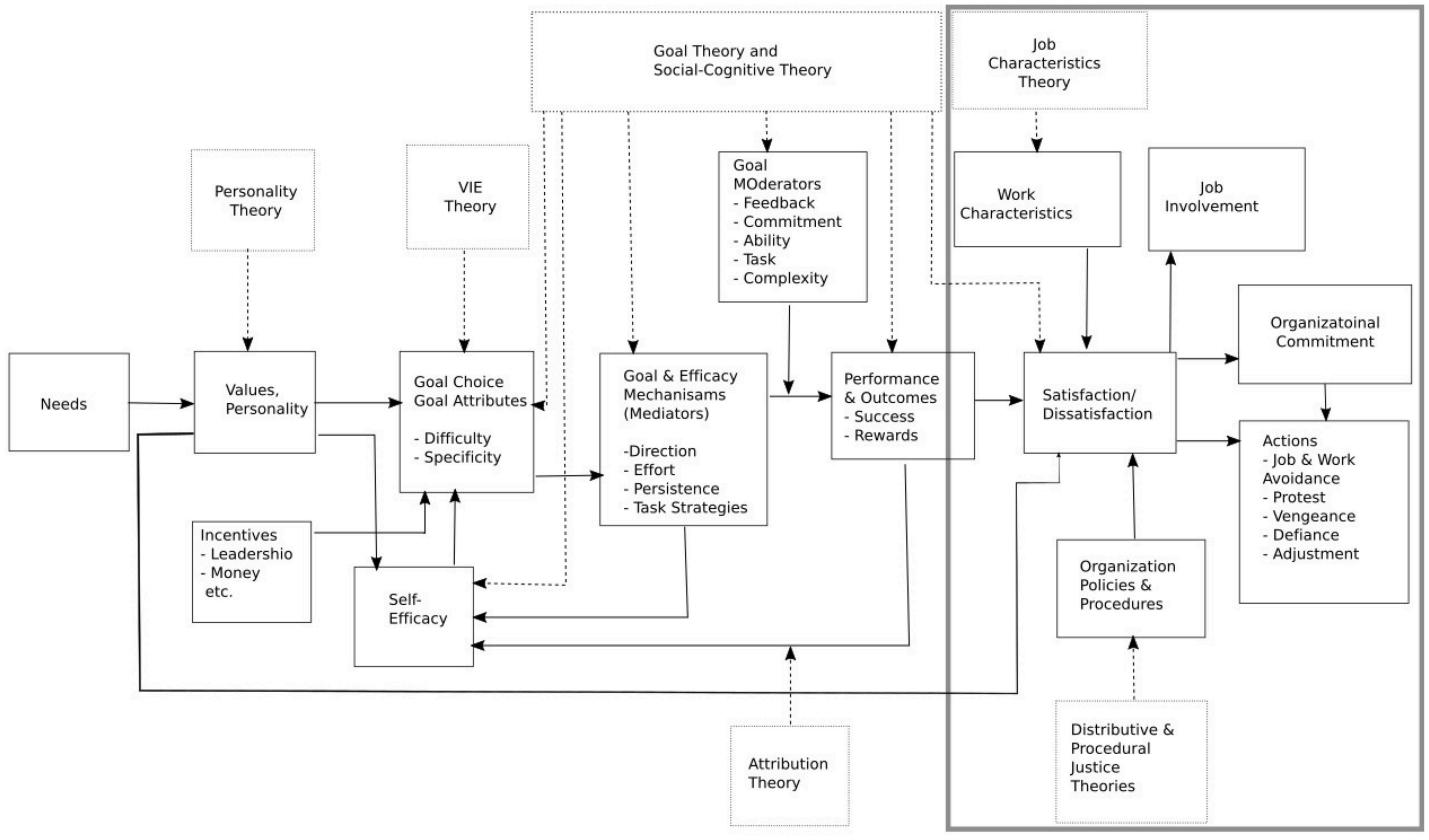
Diagram of the Latham and Locke model. The frame on the right indicates the part of the model the current study focuses on.

The model of Locke and Latham is show in Figure 1. As the figure shows, it includes individual needs, values and motive, as well as personality. Incorporating the theory of expectations, the goal-setting theory and the social-cognitive theory, it focuses on goal setting, goals themselves and self-efficiency. Performance, by way of achievements and rewards, affects job satisfaction. The model defines relations between different constructs and, in particular, that job satisfaction is affected by the job characteristics and organizational policy and procedures and that it, in turn, affects organizational commitment and job involvement. Locke and Latham suggested that the theory they proposed needs more stringent empirical validation. In the study presented here, we take a closer look at the part of their theory which addresses the relationship between job satisfaction, involvement and organizational commitment. The results of the empirical study conducted in industrial systems suggest that this part of the model needs to be improved to reflect the mediating role of job involvement in the process through which job satisfaction influences organizational commitment.

Job satisfaction is one of the most researched phenomena in the domain of human resource management and organizational behavior. It is commonly defined as a “pleasurable or positive emotional state resulting from the appraisal of oneś job or job experiences” (Schneider and Snyder, 1975; Locke, 1976). Job satisfaction is a key element of work motivation, which is a fundamental determinant of one's behavior in an organization.

Organizational commitment, on the other hand, represents the degree to which the employees identify with the organization in which they work, how engaged they are in the organization and whether they are ready leave it (Greenberg and Baron, 2008). Several studies have demonstrated that there is a strong connection between organizational commitment, job satisfaction and fluctuation (Porter et al., 1974), as well as that people who are more committed to an organization are less likely to leave their job. Organizational commitment can be thought of as an extension of job satisfaction, as it deals with the positive attitude that an employee has, not toward her own job, but toward the organization. The emotions, however, are much stronger in the case of organizational commitment and it is characterized by the attachment of the employee to the organization and readiness to make sacrifices for the organization.

The link between job satisfaction and organizational commitment has been researched relatively frequently (Mathieu and Zajac, 1990; Martin and Bennett, 1996; Meyer et al., 2002; Falkenburg and Schyns, 2007; Moynihan and Pandey, 2007; Morrow, 2011). The research consensus is that the link exists, but there is controversy about the direction of the relationship. Some research supports the hypothesis that job satisfaction predicts organizational commitment (Stevens et al., 1978; Angle and Perry, 1983; Williams and Hazer, 1986; Tsai and Huang, 2008; Yang and Chang, 2008; Yücel, 2012; Valaei et al., 2016), as is the case in the study presented in this paper. Other studies suggest that the organizational commitment is an antecedent to job satisfaction (Price and Mueller, 1981; Bateman and Strasser, 1984; Curry et al., 1986; Vandenberg and Lance, 1992).

In our study, job involvement represents a type of attitude toward work and is usually defined as the degree to which one identifies psychologically with one's work, i.e., how much importance one places on their work. A distinction should be made between work involvement and job involvement. Work involvement is conditioned by the process of early socialization and relates to the values one has wrt. work and its benefits, while job involvement relates to the current job and is conditioned with the one's current employment situation and to what extent it meets one's needs (Brown, 1996).

## 2. Methods

### 2.1. Research method

**Figure 2:**
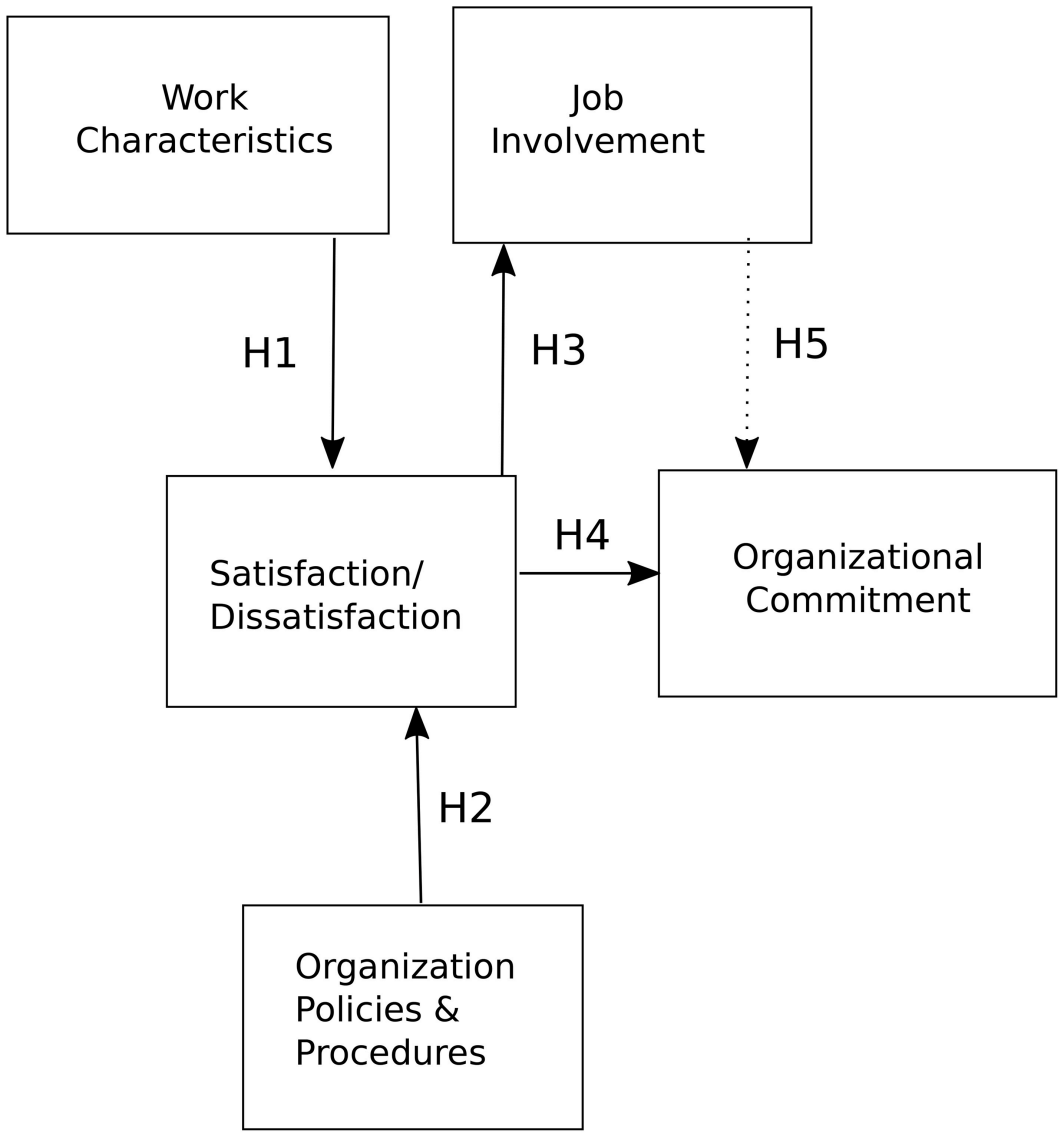
The research model.

Based on the relevant literature, the results of recent studies and the model proposed by Locke and Latham (2004), we designed a conceptual model shown in Figure 2. The model was then used to formulate the following hypotheses:

H0 - Work motivation factors, such as organizational commitment, job involvement, job satisfaction and work characteristics, represent interlinked significant indicators of work motivation in the organizations examined.
H1 - Work characteristics will have a positive relationship with job satisfaction.
H2 - Organizational policies and procedures will have a positive relationship with job satisfaction.
H3 - Job satisfaction will have a positive relationship with job involvement.
H4 - Job satisfaction will have a positive relationship with organizational commitment.
H5 - Job involvement will have a mediating role between job satisfaction and organizational commitment.

### 2.2. Participants

For the purpose of this study, 125 organizations from the Serbian Chamber of Commerce database (www.stat.gov.rs) were randomly selected to take part in this study. Each organization was contacted and an invitation letter was sent. Eight companies expressed a desire to take part and provided contact details for 700 of their employees. The questionnaire distribution process was conducted according to Dillman's approach (Dillman, 2011). Thus, the initial questionnaire dissemination process was followed by a series of follow-up email reminders, if required. After a 2-month period, out of 625 received, 566 responses were valid. Therefore, the study included 566 persons, 235 males (42%) and 331 women (58%) employed by 8 companies located in Serbia, Eastern Europe.

The sample encompassed staff from both public (53%) and private (47%) companies in manufacturing (31%) and service (69%) industries. The companies were of varied size and had between 150 and 6,500 employees, 3 of them (37.5%) medium-sized (<250 employees) and 5 (62.5%) large enterprises.

For the sake of representativeness, the sample consisted of respondents across different categories of: age, years of work service and education. The age of the individuals was between 20 and 62 years of age and we divided them into 5 categories as shown in Table 1. The table provides the number of persons per category and the relative size of the category wrt. to the whole sample. In the same table, a similar breakdown is shown in terms of years a person spent with the company, their education and the type of the position they occupy within the company (managerial or not).

**Table 1:** Data sample characteristics.

		**Frequency**	**Percent(%)**
Sex	Male	235	41.5
	Female	331	58.5
Age	20–29	127	22.5
	30–39	202	35.8
	40–49	121	21.5
	50–59	100	17.7
	60–69	14	2.5
Years with the company	0-9	262	46.3
	10–19	151	26.7
	20–29	103	18.2
	30–40	50	8.8
Education	High School	282	49.8
	Community college	59	10.4
	College	195	34.5
	Postgraduate	30	5.3
Vocation	Technical	262	46.3
	Natural sciences	56	9.9
	Humanistic sciences	248	43.8
Company ownership	Public	303	53.4
	Private	264	46.6
Company type	Production	173	30.5
	Service	394	69.5
Employee position	Non-managerial	494	87.3
	Managerial	72	12.7

### 2.3. Ethics statement

The study was carried out in accordance with the Law on Personal Data Protection of the Republic of Serbia and the Codex of Professional Ethics of the University of Novi Sad. The relevant ethics committee is the Ethics Committee of the Faculty of Technical Sciences of the University of Novi Sad.

All participants took part voluntarily and were free to fill in the questionnaire or not.

The questionnaire included a cover sheet explaining the aim of the research, ways in which the data will be used and the anonymous nature of the survey.

### 2.4. Measures

This study is based on a self reported questionnaire as a research instrument.

The questionnaire was developed in line with previous empirical findings, theoretical foundations and relevant literature recommendations (Brayfield and Rothe, 1951; Weiss et al., 1967; Mowday et al., 1979; Kanungo, 1982; Fields, 2002). We then conducted a face validity check. Based on the results, some minor corrections were made, in accordance with the recommendations provided by university professors. After that, the pilot test was conducted with 2 companies. Managers from each of these companies were asked to assess the questionnaire. Generally, there were not any major complaints. Most of the questions were meaningful, clearly written and understandable. The final research instrument contained 86 items. For acquiring respondents' subjective estimates, a five-point Likert scale was used.

The questionnaire took about 30 min to fill in. It consisted of: 10 general demographic questions, 20 questions from the Minnesota Satisfaction Questionnaire (MSQ), 15 questions from the Organizational Commitment Questionnaire (OCQ), 10 questions from the Job Involvement Questionnaire (JIQ), 18 questions of the Brayfield-Rothe Job Satisfaction Scale (JSS), 6 questions of the Job Diagnostic Survey (JDS) and 7 additional original questions related to the rules and procedures within the organization.

The Minnesota Satisfaction Questionnaire (MSQ), 20 items short form (Weiss et al., 1967), was used to gather data about job satisfaction of participants. The MSQ – short version items, are rated on 5-points Likert scale (1 very dissatisfied with this aspect of my job, and 5 – very satisfied with this aspect of my job) with two subscales measuring intrinsic and extrinsic job satisfaction.

Organizational commitment was measured using The Organizational Commitment Questionnaire (OCQ). It is a 15-item scale developed by Mowday, Steers and Porter (Mowday et al., 1979) and uses a 5-point Likert type response format, with 3 factors that can describe this commitment: willingness to exert effort, desire to maintain membership in the organization, and acceptance of organizational values.

The most commonly used measure of job involvement has been the Job Involvement Questionnaire (JIQ, Kanungo, 1982), 10-items scale designed to assess how participants feel toward their present job. The response scale on a 5-point scale varied between “strongly disagree/not applicable to me” to “strongly agree/fully applicable”.

The Brayfield and Rothe's 18-item Job Satisfaction Index (JSI, Brayfield and Rothe, 1951) was used to measure overall job satisfaction, operationalized on five-point Likert scale.

Psychometric analysis conducted showed that all the questionnaires were adequately reliable (Cronbach alpha > 0.7). The suitability of the data for factor analysis has been confirmed using the Kaiser-Meyer-Olkin (KMO) Test (see Table 2).

**Table 2:** Basic psychometric characteristics of the instruments.

**Scale**		**Suitability KMO measure**	**Reliabilty cronbach's α**
MSQ	Overall	0.936	0.924
	Extrinsic motivation	0.905	0.885
	Intrinsic motivation	0.897	0.859
OCQ	Original	0.901	0.841
Brayfield-Rothe Job Satisfaction index	Original	0.895	0.83
	Improved	0.905	0.867
Job involvement	Original	0.886	0.842
	Improved	0.886	0.854
Work characteristics	Original	0.878	0.907

For further analysis we used summary scores for the different scales. Job satisfaction was represented with the overall score of MSQ, as the data analysis revealed a strong connection between the extrinsic and intrinsic motivators. The overall score on the OCQ was used as a measure of organizational commitment, while the score on JDS was used to reflect job characteristics. The JSS and JIQ scales have been modified, by eliminating a few questions, in order to improve reliability and suitability for factor analysis.

## 3. Results

Statistical analysis was carried out using the SPSS software. The SPSS Amos structural equation modeling software was used to create the Structural Equation Models (SEMs).

The data was first checked for outliers using box-plot analysis. The only outliers identified were related to the years of employment, but these seem to be consistent to what is expected in practice in Serbia, so no observations needed to be removed from the dataset.

### 3.1. Exploratory factor analysis

Although research dimensions were empirically validated and confirmed in several prior studies, to the best of our knowledge, the empirical confirmation of the research instrument (i.e., questionnaire) and its constituents in the case of Serbia and South-Eastern Europe is quite scarce. Furthermore, the conditions in which previous studies were conducted could vary between research populations. Also, such differences could affect the structure of the research concepts. Thus, exploratory factor analysis (EFA) was conducted in order to empirically validate the structure of research dimensions and to test the research instrument, within the context of the research population of South-Eastern Europe and Serbia.

**Figure 3:**
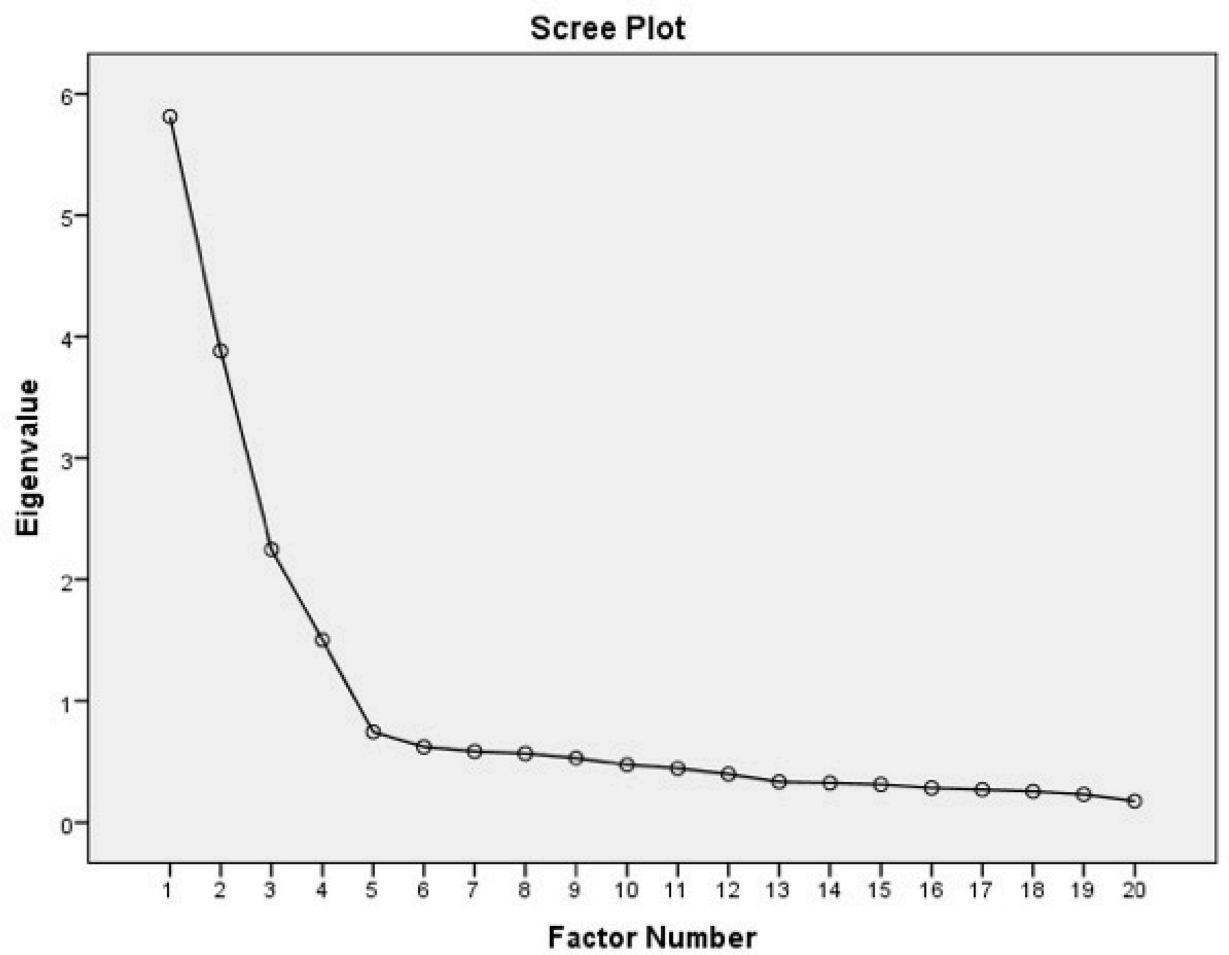
Scree plot of the EFA results.

Using the maximum likelihood method we identified four factors, which account for 67% of the variance present in the data. The scree plot of the results of the analysis is shown in Figure 3. As the figure shows, we retained the factors above the inflection point.

The communalities for the variables loading into the factors are shown in Table 3 and the questions corresponding to our variables are listed in Table 4. Initial communalities are estimates of the proportion of variance in each variable accounted for by all components (factors) identified, while the extraction communalities refer to the part of the variance explained by the four factors extracted. The model explains more of the variance then the initial factors, for all but the last variable.

**Table 3:** Communalities.

	**Initial**	**Extraction**
characteristics01	0.604	0.562
characteristics02	0.665	0.638
characteristics03	0.666	0.709
characteristics04	0.715	0.759
characteristics05	0.702	0.736
characteristics06	0.637	0.641
commitment02	0.537	0.659
commitment06	0.549	0.647
commitment10	0.431	0.47
commitment14	0.431	0.445
satisfaction14	0.586	0.631
satisfaction16	0.577	0.623
satisfaction15	0.578	0.629
satisfaction04	0.559	0.593
satisfaction11	0.446	0.457
involvement05	0.599	0.703
involvement04	0.554	0.639
involvement03	0.418	0.457
involvement08	0.445	0.412
involvement09	0.416	0.401

**Table 4:** Questions that build our constructs.

**Job characteristics**	
characteristics01	Stimulating and challenging work.
characteristics02	Chances to exercise independent thought and action.
characteristics03	Opportunities to learn new things from my work.
characteristics04	Opportunities to be creative and imaginative in my work.
characteristics05	Opportunities for personal growth and development.
characteristics06	A sense of worthwhile accomplishment in my work.
**Job involvement**	
involvement03	I am very much involved personally in my job.
involvement04	I live, eat and breathe my job.
involvement05	Most of my interests are centered around my job.
involvement08	Most of my personal life goals are job-oriented.
involvement09	I consider my job to be very central to my existence.
**Organizational commitment**	
commitment02	I talk up this organization to my friends as a great organization to work for.
commitment06	I am proud to tell others that I am part of this organization.
commitment10	I am extremely glad that I chose this organization to work for over others I was considering at the time I joined.
commitment14	For me this is the best of all possible organizations for which to work.
**Minnesota satisfaction questionnaire**	
satisfaction04	The chance to be “somebody” in the community.
satisfaction11	The chance to do something that makes use of my abilities.
satisfaction14	The chances for advancement on this job.
satisfaction15	The freedom to use my own judgment.
satisfaction16	The chance to try my own methods of doing the job.

More detailed results of the EFA for the four factors, are shown in Table 5. The unique loadings of specific items measured with the different questions in the questionnaire on the factors identified are shown in the pattern matrix (Table 6). As the table shows, each factor is loaded into by items that were designed to measure a specific construct and there are no cross-loadings. The first factor corresponds to job characteristics, second to job satisfaction, third to job involvement and the final to organizational commitment. The correlation between the factors is relatively low and shown in Table 7.

**Table 5:** Total variance explained by the dominant factors.

**Factor**	**Initial eigenvalues**			**Extraction sums of squared loadings**			**Rotation sums of squared loadings**
	**Total**	**% of variance**	**Cumulative %**	**Total**	**% of Variance**	**Cumulative %**	**Total**
1	5.815	29.075	29.075	5.220	26.102	26.102	4.268
2	3.883	19.414	48.490	3.716	18.580	44.681	4.078
3	2.248	11.241	59.731	1.786	8.932	53.614	3.405
4	1.503	7.517	67.248	1.088	5.438	59.051	3.505

**Table 6:** Pattern matrix for the factors identified.

	**Factor**			
	**1**	**2**	**3**	**4**
characteristics04	0.872			
characteristic05	0.856			
characteristic03	0.848			
characteristic02	0.796			
characteristic06	0.793			
characteristic01	0.735			
satisfaction16		0.815		
satisfaction15		0.806		
satisfaction04		0.778		
satisfaction14		0.743		
satisfaction11		0.611		
involvement04			0.808	
involvement05			0.801	
involvement09			0.667	
involvement03			0.662	
involvement08			0.617	
commitment02				0.841
commitment06				0.787
commitment10				0.672
commitment14				0.602

**Table 7:** Factor correlation matrix.

**Factor**	**1**	**2**	**3**	**4**
1	1	0.226	0.131	0.073
2	0.226	1	0.351	0.515
3	0.131	0.351	1	0.413
4	0.073	0.515	0.413	1

### 3.2. Confirmatory factor analysis

In the next part of our analysis we used Structural Equation Modeling to validate and improve a part of the model proposed by Locke and Latham (2004) that focuses on work characteristics, job satisfaction, organizational commitment and job involvement.

**Figure 4:**
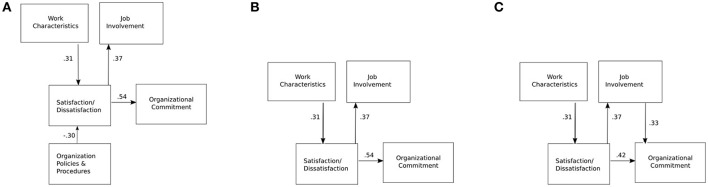
The evolution of our model (the path coefficients are standardized): **(A)** the initial model based on Locke and Latham (2004), **(B)** no partial mediation, and **(C)** partial mediation introduced.

Although the EFA suggest the existence of four, not five, dominant factors in the model, diverging from the model proposed by Locke and Latham (2004), in our initial experiments we used their original model, shown in Figure 4A, taking into account also organizational policies and procedures.

In this (default) model, the only independent variable are the job characteristics. The standardized regression coefficients shown in Figure 4A (we show standardized coefficients throughout Figure 4) indicate that the relationship between the satisfaction and organizational commitment seems to be stronger (standard coefficient value of 0.54) than the one between satisfaction and involvement (standard coefficient value of 0.37). The effect of job characteristics and policies and procedures on the employee satisfaction seems to be balanced (standard coefficient values of 0.31 and 0.30, respectively).

The default model does not fit our data well. The Comparative Fit Index (CFI) for this model is 0.759, the Tucker-Lewis Index (TLI) is 0.598, while the Root Mean Square Error of Approximation (RMSEA) is 0.192.

A more detailed analysis of the model revealed that it could indeed (as the EFA suggests) be improved by eliminating the organizational policies and procedures variable, as it has a high residual covariance with job involvement (−3.071) and organizational commitment (−4.934).

We therefore propose to eliminate the “Organizational policies and procedures” variable from the model. Dropping the variable resulted in an improved model shown in Figure 4B. The improved model fits the data better, but the fit is still not good (*RMSEA* = 0.125, *CFI* = 0.915 and *TLI* = 0.830).

We then hypothesized that job involvement influences organizational commitment, yielding the final model tested in this study (Figure 4C). This model turned out to be the one that fits our data very well (*RMSEA* = 0.000, *CFI* = 1 and *TLI* = 1.015).

## 4. Mediation analysis

In the final part of the study we conducted the mediation analysis, to understand the relationship between job satisfaction, job involvement and organizational commitment. We used bootstrapping, based on 5000 samples and the confidence interval of 95%.

**Figure 5:**
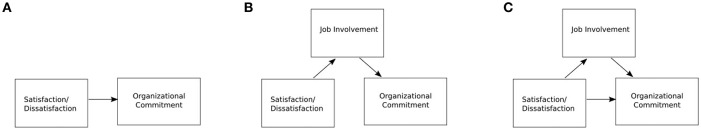
Mediation analysis models. **(A)**, Model 1; **(B)**, Model 2; **(C)**, Model 3.

We started with a model that contains just one relation between satisfaction and commitment (Figure 5A), then tested for full mediation (Figure 5B) and finally partial mediation as indicated in out proposed model (Figure 5C). The unstandardized, direct effect regression weights and the *p*-values obtained in these experiments are shown in Table 8. As the *p*-values show, all the connections in our three models are significant and that they remain so throughout the evolution of the model. Therefore, job involvement mediates the influence of satisfaction on organizational commitment, but this is a partial mediation and a major part of the effect of satisfaction on the organizational commitment is achieved directly.

**Table 8:** Mediation analysis regression weights.

	Parameter^*^	**Estimate**	**Lower**	**Upper**	p^**^
Model 1	Organizational commitment < — Satisfaction	0.47	0.363	0.586	0.000
Model 2	Organizational commitment < — Satisfaction	0.5	0.378	0.629	0.000
	Organizational commitment < — Job involvement	0.32	0.25	0.397	0.000
Model 3	Job involvement < — Satisfaction	0.472	0.352	0.601	0.000
	Organizational commitment < — Satisfaction	0.368	0.263	0.487	0.000
	Organizational commitment < — Job involvement	0.189	0.124	0.255	0.000

* , Unstandardized, direct effects; p

** *, statistically significant at 0.01*.

## 5. Discussion

We conducted an empirical study aimed at exploring the relationship between employee satisfaction, job involvement, organizational commitment, work characteristics and organizational policies and procedures.

Based on the relevant scientific literature, recent studies in the area and the integrative model of work motivation of Locke and Latham (2004), we have formulated an initial conceptual model for our research and hypothesized the connections between the relevant variables. The initial model has been improved iteratively, with the goal of increasing its fit to the empirical data collected in the study.

Starting from the model proposed by Locke and Latham (2004) we determined that their model does not fit our experimental data well and that we observe a connection between job involvement that is not present in their model. In addition, our data does not support the hypothesis that organizational procedures and policies affect employee satisfaction in the organizations considered. As a result we propose a 4 factor model shown in Figure 4C for the relationship between the concepts of work characteristics, employee satisfaction, job involvement and organizational commitment.

We analyzed the results of the study based on 1 general and 5 specific hypotheses. The research confirms that there is a link between work characteristics and job satisfaction (H1), but that it is weak, suggesting that a dominant effect of the material factors of motivation exists.

We have also determined that there is a connection between the *rules and procedures* variable (H2) and the rest of the variables, indicating that it should be considered in future studies, but that the constructs need to be operationalized better.

The third specific hypothesis (H3) that job satisfaction has a positive relationship with job involvement has been confirmed and we have observed that extrinsic work motivation has a stronger effect than intrinsic, which can be explained by low wages and insufficient funds for everyday life. Other research has confirmed this link (Govender and Parumasur, 2010) and showed that most of the employee motivation dimensions have significant links with the dimensions of job involvement (9 out of 10 pairs).

The fourth specific hypothesis (H4 - Job satisfaction will have a positive relationship with organizational commitment) has also been confirmed and we can conclude that a positive relationship exists, which is in line with recent research in this area. The subscale focused on identification with the organization is strongly connected with both intrinsic and extrinsic factors of job satisfaction, but this cannot be said for the subscale focused on organizational attachment. Our research supports the existence of a weak connection between job satisfaction and organizational attachment, both when intrinsic and extrinsic satisfaction is considered as a motivator. A study of work motivation and organizational commitment conducted in Bulgaria (Serbia's neighbor) showed that extrinsic factors are key sources of organizational commitment (Roe et al., 2000), as well as that job involvement and the chances for the fulfillment o higher-order needs pay a very important part in the motivation of the employees.

One of the reasons for such a result can be the economic situation in Serbia, which has a severely detrimental effect on work motivation. The transition and economic crisis is accompanied by the shrinking purchasing power of the population, higher unemployment rates and a rising disparity in the salary levels, all of which causes the adjustment of the behavior of the employees to these conditions. Under the economic conditions that exist in Serbia it is to be expected that the individuals will put more value on the salary and advancement prospects than on the opportunities for growth and development, which do not present a direct financial benefit.

The research did not reveal any differences with respect to the sex of the participants, regardless of the variable considered. Other research has not reached a consensus on the matter, as a part of the studies suggests that there are differences in job involvement between men and women (Lodahl and Kejnar, 1965; Hall and Mansfield, 1975; Rabinowitz and Hall, 1977; Saal, 1978).

Regarding the ownership of the organizations examined, the research revealed statistically significant differences between the employees working in public and private companies, i.e., that the participants working in the private sector scores significantly higher on every variable except work characteristics, meaning that they are more committed to work, more involved and more satisfied.

In addition, we have determined that there are statistically significant differences when it comes to the position of the employees in the organization's hierarchy, i.e., whether they occupy a managerial or a non-managerial position. The study shows that managers have higher scores for organizational attachment, organizational commitment, intrinsic motivators, extrinsic motivators, job satisfaction and job involvement. We can, therefore, conclude that the managers are more satisfied with their work in general and that they are more committed to the organization than other employees. This can be explained by the fact that, due to the nature of the work they do, they are able to make decisions, they have a more varied job and have better material and non-material rewards. A more detailed analysis of the commitment of the managers, focused on identifying if we are dealing with normative, continuous or affective commitment would provide more insight into the structure and nature of the relationship between the organization and the individual.

Considering the type of the company (manufacturing or service) our study showed that the participants working in manufacturing companies are the ones who identify more with the company, are more committed to the company, more satisfied with their work and more involved.

Our study also identified a significant difference with respect to the vocation of the participants, showing that those with training in humanistic sciences awarded most positive scores to the characteristics of their work, while the opposite was true for those of technical vocations.

The part of our analysis focused on the age of the participants revealed that there is a statistically significant connection between the age and job satisfaction, where the older the employee, the less satisfied he/she is with their job and cares less about the characteristics of work. A reason for such a result could again be found in the economic situation of Serbia and the high unemployment rate (over 20%), causing the younger people to be satisfied with the simple fact that they managed to get a job, rather than being satisfied with the job itself. Another reason could be the difference in the perception of desires and possibilities that exists between the younger and older employees.

The years with the company are negatively linked with employee satisfaction, as well as job characteristics, which is in line with the effect discussed in the previous paragraph, as those with more time spent in the company are less satisfied with their job and care less about the characteristics of their work.

Considering the level of education of the participants, our study showed that the more educated the employees are, the less involved they are in their work and that they seem to care more about the characteristics of their work.

Our research showed that links exist between all the variables studied and that the weakest of these links is between work characteristics and other constructs. Of those, the weakest link in turn is the link between the work characteristics and the subscale of organizational commitment related to the identification with the organization. Thus, we can conclude that work characteristics do not exhibit a significant influence on whether and to what extent the employee will identify with the organization in which he/she works, i.e., whether he/she will be committed to the organization.

A moderate to strong connection exists between organizational commitment and job satisfaction, which is in line with the results of numerous previous studies (Currivan, 1999; Meyer et al., 2002; Malhotra and Mukherjee, 2004; Saari and Judge, 2004; Chen, 2007; Falkenburg and Schyns, 2007; Moynihan and Pandey, 2007; Getahun et al., 2008; Colakoglu et al., 2010; Yücel, 2012; Fu and Deshpande, 2014).

Our study confirms the existence of a strong connection between job satisfaction and job involvement (Moynihan and Pandey, 2007; Wegge et al., 2007; Griffin et al., 2010; Raymond and Mjoli, 2013; Zopiatis et al., 2014). Many studies have been carried out in an attempt to examine and define the relationship between job involvement and organizational commitment. Our results are in line with previous studies, which diverge only on the strength of the connection, ranging from moderate to strong (Blau and Boal, 1989; Brewer and Lok, 1995; Sjöberg and Sverke, 2000; Brooks and Swailes, 2002; Toga, 2011). Our study provides more evidence for the existence of such a relationship, which is moderately strong. Such a relationship does not exist in the integrative model of Locke and Latham (2004), which served as a starting point for this study.

In addition, we have determined that job involvement has a mediating role between job satisfaction and organizational commitment. Job involvement mediates the influence of satisfaction on organizational commitment, but this is a partial mediation and a major part of the effect of satisfaction on the organizational commitment is achieved directly.

The construct related to organizational policies and procedures seems not to have significant bearing on employee satisfaction, based on the data collected. Two plausible explanations exist for this. The first is the fact that this was the only construct in our study for which a suitable standard questionnaire could not be found, so one had to be constructed specifically, meaning that the construct should be operationalized better in future studies and that this represents the limitation of our study. The other is the fact that in Serbia, as in most transition economies, the lack of suitable institutional and legislative framework at the national level is often accompanied with lax, not clearly defined and even less adhered-to business policies and procedures. In such a state of affairs, the employees seldom have a relatively clear idea of what the policies and procedures of their organization are and are unable to evaluate them with respect to those of other organizations, making this construct very hard to measure. At the same time it can be argued that, in such a situation, the policies and procedures are not perceived by the employees as a significant factor of their organizational behavior and indeed do not affect their work motivation. Whatever the reason, the relationship of policies and procedures to the other variables of work motivation within the transition economies merits further investigation.

## Author contributions

JĆ and SM designed the study. JĆ collected the data and conducted the bulk of the research. MD and DĆ conducted the statistical analysis and modeling. All authors took part in the manuscript writing, led by JĆ and DĆ.

### Conflict of interest statement

The authors declare that the research was conducted in the absence of any commercial or financial relationships that could be construed as a potential conflict of interest.
